# An analysis of entity normalization evaluation biases in specialized domains

**DOI:** 10.1186/s12859-023-05350-9

**Published:** 2023-06-02

**Authors:** Arnaud Ferré, Philippe Langlais

**Affiliations:** 1 MaIAGE, INRAE, Université Paris-Saclay, Jouy-en-Josas, France; 2 RALI, DIRO, Université de Montréal, Montreal, Canada

**Keywords:** Entity normalization, Evaluation, Dataset, Corpus, Ablation study

## Abstract

**Background:**

Entity normalization is an important information extraction task which has recently gained attention, particularly in the clinical/biomedical and life science domains. On several datasets, state-of-the-art methods perform rather well on popular benchmarks. Yet, we argue that the task is far from resolved.

**Results:**

We have selected two gold standard corpora and two state-of-the-art methods to highlight some evaluation biases. We present non-exhaustive initial findings on the existence of evaluation problems of the entity normalization task.

**Conclusions:**

Our analysis suggests better evaluation practices to support the methodological research in this field.

## Background

### Introduction

Entity normalization (EN), also known as concept normalization/grounding or entity linking/disambiguation/grounding, is a challenging information extraction task. Interest for this task emerged in the biomedical field in the early 2000s [1]. Nowadays, biomedical annotated corpora still represent the majority of the gold standards for evaluation of EN methods [2–4]. Our focus in this article is to show potential biases of existing evaluation benchmarks, and to make recommendations for better evaluation practices.

#### Definition of the entity normalization task

**Fig. 1:**
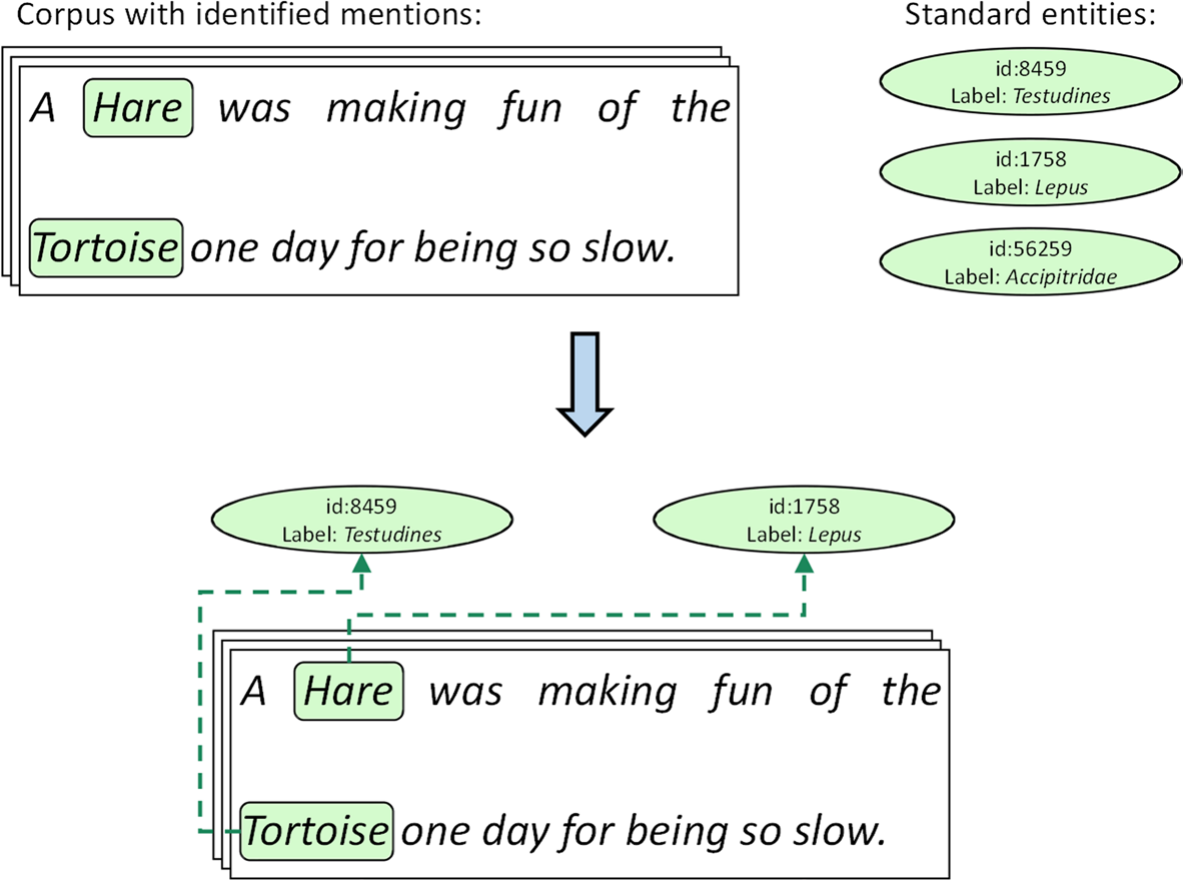
An example of a small entity normalization task. The goal is to link animal mentions extracted from texts to a standard entity from an available set of unambiguous references. Standard entities have only one label which represents the scientific name of an animal and differ here from the vernacular names used in text

*The goal of EN is to link identified entity mentions to standard entities from an available set of unambiguous references (ontology, terminology, thesaurus, dictionary, …)* (see Fig. 1). The entity mentions are possibly represented by multi-word non-contiguous expressions, for which it is known that they are of interest for the task (e.g. animal mentions). This task commonly assumes an entity recognition step firstly extracts the mentions (i.e. mention detection/extraction) and determines whether or not they are of interest to the task, that is, to classify them in some entity type of interest or not. The normalization task then consists in linking these identified mentions of interest to zero, one or several standard entities. Zero is for mentions that should be normalized by a standard entity of interest, but which is absent from the set of references. These cases are known as not-in-lexicon/not-in-list (NIL) cases or out-of-KB. Cases where more than one standard entity is possible are referred to as multi-normalization. For instance, a mention such as “*pasteurized milk cheeses*” which may be normalized by concepts <cheese> and <pasteurized food> (sometimes called a multi-labeled mention), or an unprocessed composite mention such as “*breast or ovarian cancer*” which may be normalized by concepts <Breast Neoplasms> and <Ovarian Neoplasms>. The set of references often takes the form of an ontology or a knowledge base (or can often be related to it), and each standard entity is therefore a concept or an instance, that is a unique identifier and one or many associated textual labels (e.g. a preferred term, some synonyms). To standardize the vocabulary used in the remainder of this article, we mainly use the “*ontology*” (as set of references) and “*concept*” (as standard entity) terms, which fit easily with the addressed datasets.

#### Main difficulty: terminological variation

EN in specialized domains is mainly concerned with the problem of terminological variations, that is, mentions with distinct surface forms that point to the same concept [5, 6]. Moreover, a mention should be sometimes normalized by a concept whose no label matches this surface form [7]. For instance, 60% of the mentions from the Bacteria Biotope dataset from the BioNLP 2013 Shared Task have a surface form different from the labels of the concept which should normalize them. The main source of variations are inflection (e.g. the mention “*tortoises*” which could be normalized by a concept <tortoise>), synonymy (e.g. “*chelonian*”), hyponymy (e.g. “*Testudo graeca*”), nominal expansion (e.g. “*georgia populations of gopher tortoises*”) and morphosyntactic variation (e.g. “*gopher tortoises populations in Georgia*”).

#### Terminological statement: difference between entity normalization and entity disambiguation/linking

In many ways, entity disambiguation shares similarities to entity normalization. As noticed in [8], the names of several tasks related to entity linking still seem to be vaguely defined. For instance, some consider that “*entity linking*” refers to the overall task of entity recognition and entity disambiguation [9], while others consider that it is similar to entity disambiguation [10]. Moreover, some consider entity linking and entity normalization as synonyms [11, 12]. We will consider in this article that both normalization and disambiguation are subtasks of entity linking. So what is the difference? We believe that in open domains, a basic matcher between labels and mentions gives many concept-candidates for each mention, and the main focus is then to select the unique correct concept among those candidates [10, 13]. For instance, simple heuristics would match the surface form of a mention “*paris*” to numerous titles of Wikipedia pages, but it is more difficult to find if this mention points to a city, a person, a movie, etc. Moreover, entity disambiguation deals mainly with named entities, while entity normalization deals mainly with terminological variations. In a similar way, semantic categories are more often concepts in specialized domains, where there are mainly instances in open domains (e.g. persons, localizations, organizations). Finally, the reference vocabulary in open domains is often based on Wikipedia data [14, 15], resulting in the availability of descriptions for each category (i.e. each Wikipedia article is considered as a reference, and its title as a label), which can be used as a supplementary information. These descriptions remain rare in ontological references developed in specialized domains. Nevertheless, some disambiguation tasks propose tracks where the use of these descriptions are not allowed [16].

#### How is EN commonly addressed?

To our knowledge, methods can be mainly characterized by four types of strategies: *dictionary-based* (DictB), *distributional representations* (DistR), *symbolic rules* (SymR) and *machine learning* (ML), that can be combined. Historically, the first approach [1, 12] was a linguistic approach based on dictionaries, association measures, morphological and/or syntactic properties of texts [6], and which mainly aimed to find a match between the mention’s surface form and a label’s surface form of a concept. If a match is found, then the concept that has the label is predicted for the mention. That type of approach is often referred to as dictionary-based (DictB), or sometimes as lexicon-based. This is often done by increasing the possibilities of matching (e.g. lemmatize the surface forms or increase the number of labels associated with the references) while trying to deal with the potentially created ambiguities (e.g. collapsing two semantically different mentions/labels by applying stemming). Rather than using surface forms, another approach is based on distributional representations (DistR) of words and multi-words expressions, with the aim to deal with terminological variations. In that way, two different surface forms with a similar meaning can have close vector representations. Concurrently, normalization methods can use symbolic rules (SymR) or machine learning (ML) tools, whether it uses surface forms or distributional representations. For the ML approach, we define here a training example as a pair [mention, concept(s)], which allows us to define normalization as a multiclass classification of mentions (possibly multi-output when multi-normalization cases are possible).

To address normalization, some preprocessing of input data is typically applied. Such preprocessing seems to have gained little interest in the community, even if the few articles describing ablation studies show significant impacts on results [11, 17, 18]. We can broadly categorize the preprocessing into two types: domain-specific (e.g. ambiguous word blacklist, typo resolution) and generic (e.g. lowercasing, lemmatization, stopword and punctuation removal).

Most recent NLP methods, based on deep learning, require heavy computing resources, which limits their applications when limited computing facilities are available. In addition, they have a significant ecological impact and invite the production of less greedy methods [19]. As explained recently in [11, 20], some competitive EN methods are based on BERT models, which are indeed computationally expensive. They each propose a “lightweight” DistR-ML method, but speed of calculation is only observed at inference time and on professional computing infrastructures.

Finally, several add-on strategies have emerged in order to integrate more information to methods, with the aim to address the common scarcity of data in normalization tasks. For instance, Ferré et al. [21] is the first work to integrate subsumption knowledge from the ontology in concept representations with some success. More recently, Pattisapu et al. [22] proposed a variant using existing tools to build dense concept representations from ontological graphs. A weak supervision strategy, that is, a strategy based on the production of non hand-labeled training data (i.e. of lower quality) can be used too by ML-based methods. The usual way is to augment training data by using each label of concepts (e.g. the label “*testudines*” from the concept identified by the unique identifier id:8459) as a mention, and the associated concept (e.g. the concept id:8459) as the category to predict [17, 23].

#### How is EN evaluated?

##### Evaluation based on manually annotated corpora

To evaluate and compare normalization methods, some corpora manually annotated with ontology concepts have been created. In all cases, these corpora are the result of annotations by domain experts on texts where the boundaries of the mentions of interest are identified, and for which concept(s) from one or many chosen ontologies are associated. To be able to compare results fairly, an annotated corpus is then separated into at least two parts: a training and a test set. The training set is used to learn and optimize parameters of methods. The trained method is then used to do predictions on the test set. The difference between predictions and annotations on the test set enables to estimate the performance of a method. Frequently, a third set is further produced, called the development set. This one enables method developers to estimate the generalization capacity of their method, while limiting overfitting on the test set [24, 25].

To limit some dataset overfitting and thus have a fair estimation of how accurate a method is, k-fold cross-validation techniques have emerged. It mainly results in a separation into k folds of train(+ development) + test sets [6, 26]. The final score of a method is then the average of the scores on the k sub-sets.

##### Evaluation measures

The consensus evaluation metric used at the task level is the accuracy, which is basically the average of a strict metric over all evaluated mentions (see Eq. 1). More recently, less strict metrics have been introduced in normalization evaluations, such as top-k accuracy or similarity scores. Top-k accuracy (see Eq. 2) can be used when a method ranks at least k candidates for each mention, which is quite common with DistR-based methods. If the top-k accuracy of a method is high, a database curator could validate annotations more quickly by inspecting only k concept proposals (i.e. not all the concepts of the ontology), among which there is a great probability that the correct concept is. This might save time during database curation.

1 $$Acc = \frac{1}{N} \cdot \mathop \sum \limits_{i = 1}^{N} strict\left( {m_{i} ,c_{i} } \right),$$

where $strict\left( {m,c} \right) = 1$ if *c* is the correct concept of *m*, 0 otherwise.

Equation 1: General equation of accuracy. Here $c_{i}$ is the predicted concept for the mention $m_{i}$, and *N* is the number of mentions in the dataset.

2 $$Acc@k = \frac{1}{N} \cdot \mathop \sum \limits_{i = 1}^{N} strict\left( {m_{i} ,\left\{ {c_{i}^{1} , \ldots ,c_{i}^{k} } \right\}} \right),$$

where $strict\left( {m,\left\{ {c^{1} , \ldots ,c^{k} } \right\}} \right) = 1$ if one of the concepts in $\left\{ {c^{1} , \ldots ,c^{k} } \right\}$ is the correct concept of *m*, 0 otherwise.

Equation 2: General equation of accuracy. Here $\left\{ {c^{1} , \ldots ,c^{k} } \right\}$ is the predicted k most ranked candidates.

Also, a similarity score uses the ontological graph (when available) to calculate a non-strict score by calculating a similarity distance between the predicted concept and the correct one. The objective is to evaluate the overall severity of errors, which means that a wrongly predicted concept may get some reward proportional to its distance to the correct concept. Wright [27] introduces the Lowest Common Ancestor distance and compares the result of their method with this score and the accuracy score. A possible limitation of this distance is that it does not take into account the depth of the concepts in the ontology, which could however allow a more accurate estimation of semantic similarities between concepts. Overcoming this limitation, Maynard et al. [28] proposes to use the “*learning accuracy*” measure [29], and all the Bacteria Biotope datasets from 2013 [7] used the measure from [19].

#### Threats for validity

Identifying biases in an evaluation protocol is a key path to meaningful comparisons of methods. In the following, we formulate a number of sources of biases we have identified and that we seek to illustrate. For this, we report several experiments we conducted to demonstrate the existence of these biases through two popular gold standards. We also used two state-of-the-art methods, and a custom baseline method.

##### H1: uncertain method robustness on dataset variability

It is expected that the performance of a method on a dataset is similar to that on any corpus of the same specific domain, type of texts and language. In practice however, small variations in experimental conditions often yield to drastic changes in performance. While overclaiming is not the rule, some authors may be over optimistic about the generalization of their approach. For instance, in [23], the authors wrote “*Our work is generally applicable to any type of entity*”, even if in most cases, authors tend to delimit the range of applicability of their method. For instance, some methods are assumed as ontology-specific, such as MetaMap: “*[…] providing access to the concepts in the unified medical language system (UMLS) Metathesaurus from biomedical text*” [30]. Others delimit the type of text and the domain, such as in [31]: “*we deal with medical concept normalization in user generated texts*”.

As a consequence, to estimate the robustness of their method, more and more authors evaluate it on several datasets. For instance, Sung et al. [23] presents: “*our model […] consistently outperforms previous state-of-the-art models almost reaching the upper bound on each dataset*”. But even in that case, what do multiple evaluations demonstrate? If several gold standards share common features, a method could greatly perform on them, but not at all on distinct others. By the way, to our knowledge, there is no study on the feature similarities or dissimilarities of these used datasets, and their impact on robustness. And yet, it seems that the datasets from used batches often have relatively similar domains and/or types of documents. Moreover, datasets are often created with some simplifications compared to realistic normalization tasks. For instance, NIL and multi-normalization cases can be removed [32] or training examples can be adapted to better fit the associated test set, as the opposite is considered as a challenging task for ML approaches.

##### H2: scoring metrics bias

If there is no online evaluation platform or independent evaluation programs, authors compute the scores for their methods by themselves. Thus, it is very likely that everyone does not use the exact same scoring function, which is disturbing. In entity linking, Röder et al. [33] starts from this observation to evoke that: "*this heterogeneous landscape of […] measures leads to a poor repeatability of experiments, which makes the evaluation of the real performance of novel approaches against the state-of-the-art rather difficult*”.

##### H3: unclear description about the resources/processing used

A method is the assembly of some components that take some resources as input. However, some components may receive more attention in an article (e.g. a new neural architecture) at the expense of others (e.g. data preprocessing) without regard for what really matters in the end. Moreover, different resources could be used (e.g. a handmade typo resolution list). The threat for validity would not be a performance issue, but a poor description of what actually has a significant contribution to the performance of the method. As a result, for instance, one whole method could perform better than another one, but only through preprocessing, which could itself be used for a specific method for a performance gain. If such preprocessing and its contribution is poorly analyzed, there is a possible bias on what is really at the source of the effectiveness of the associated method. This also can cause reproducibility issues (see “H4: reproducibility issues” section).

##### H4: reproducibility issues

To validate the effectiveness of a method, it should be possible to reproduce its results independently. This becomes difficult if the method is not fully described, if its code is not shared and usable, or if a used gold standard is not available.

### Related works

#### Gold standards overview

For entity normalization in specialized domains, we present popular normalization datasets whose main features are summarized in Table 1. Entity normalization is not addressed as much as entity recognition or relation extraction. Maybe partly because it implies detailed and specialized references, which implies a great deal of effort on the part of experts in the field. As a consequence, there are not many gold standard datasets, and most of them are in English. In some cases, the normalization is not evaluated as a separate task but jointly with an entity recognition task [34], which limits the estimation of performance specifically on it (i.e. the errors on recognition are propagated on normalization). For this reason, we limited our study to datasets for the normalization task alone. There are also some disambiguation datasets in open domains of which the most used seems to be the AIDA-CoNLL-YAGO dataset [35], but as stated above, the difference of difficulties and approaches between disambiguation and normalization has led us to not consider these datasets in this work. Note that unlike open domains, even if local and global contexts of extracted mentions are often available, the majority of existing methods use only the surface forms of mentions.

**Table 1:** Summary of the 11 EN datasets presented

	Text	Ontology	Ontology size	Publicly available?
BB-norm 2019	Scientific literature	OntoBiotope	Small	Yes
NCBI-DC	Scientific literature	MEDIC	Small	Yes
BC5CDR-D	Scientific literature	MEDIC	Small	Yes
BC5CDR-C	Scientific literature	CTD-Chemical	Big	Yes
Custom CADEC	Social media	“Clinical Finding” from SNOMED-CT + AMT	Big*	Partially**
COMETA	Social media	SNOMED-CT	Big	Yes (SNOMED-CT from the UMLS)
PsyTAR	Social media	SNOMED-CT + UMLS	Big*	Partially**
SMM4H 2017	Social media	MedDRA	Intermediary	Yes
TwADR-L	Social media	SIDER 4 database of drug profiles	Small	Yes
TAC-ADR 2017	Drug labels	MedDRA	Intermediary	Yes^a^
ShARe/CLEF	Clinical report	Disorder semantic group from the UMLS	Intermediary	No
MCN	Clinical report	SNOMED-CT + RxNorm	Big	Yes

We categorize as small an ontology with less than 10,000 concepts, intermediary with between 10,000 and 100,000 concepts, and big with more

^*^Note that on some corpora, due the availability of the test set, methods are sometimes evaluated on a subpart of the ontology, although the range of entities to extract do not invite to this

^**^The annotated corpus is openly available, but not the specific version of the ontologies

^a^Test set is publicly available at: https://github.com/tigerchen52/Biomedical-Entity-Linking/tree/master/output/adr

##### BB-norm dataset of bacteria biotope 4 from BioNLP OST 2020

The BioNLP Open-Shared Tasks^1^ proposed several datasets, including 6 tasks on the Bacteria Biotope 4 corpus [2], of which the “*BB-norm*” normalization task. This task can be separated into three distinct normalization subtasks, which are evaluated separately: normalization of microorganisms by the NCBI Taxonomy [36], normalization of bacterial habitats by the biotopes part of the OntoBiotope ontology^2^ [37], and normalization of phenotypes by the phenotypes part of the same OntoBiotope ontology. The dataset is separated into training/development/test sets, but the test annotations are not provided. The evaluation is done by an online platform^3^ by uploading the predictions. This dataset is based on the entity “*categorization dataset*” of Bacteria Biotope 3 from BioNLP Shared Task 2016 [24]. For instance, similarly to the example in Fig. 1, the mention “*populations of gopher tortoises*” should be normalized by the Ontobiotope concept OBT:001351 (with label “*Tortoise*”), because a tortoise is in particular a bacterial habitat.

##### The NCBI Disease Corpus (NCBI-DC)

The NCBI Disease Corpus [25] is an annotated corpus divided into training/development/test sets. All the disease mentions have been normalized in 793 PubMed abstracts by disease concepts from MEDIC (MErged DIsease voCabulary). MEDIC [38] is the result of merging the Medical Subject Headings^4^ (MeSH) and the Online Mendelian Inheritance in Man^5^ (OMIM) ontologies, resulting in 9661 disease concepts. A particularity of this corpus is the presence of few rare unresolved composite mentions (e.g. “*breast or ovarian cancer*”), which should be normalized by the conjunction of several individual constituents concepts (e.g. <Breast Neoplasms> and <Ovarian Neoplasms>).

##### The CSIRO adverse drug event corpora (CADEC)

There are nowadays three CADEC datasets. The initial corpus [39] is the result of the annotation of posts from the medical forum AskaPatient.^6^ The annotated mentions are drugs, adverse effects, symptoms and diseases. Two ontologies are used: the non-hierarchical Australian Medicines Terminology (AMT) for drug names and the “*Clinical Finding*” subpart of the Systematized Nomenclature of Medicine Clinical Terms (SNOMED CT) for all the rest. Both ontologies have licency issues.^7^ Another version uses the Medical Dictionary for Regulatory Activities^8^ (MedDRA). To compare approaches, Limsopatham and Collier [26] proposed a new dataset separated in 10 folds train/dev/test, now frequently named “*Random CADEC*”^9^ by the community (sometimes also named AskaPatient). The dataset does not come along with the ontologies, and researchers evaluate their methods on this corpus by using only the list of the 1036 concepts used to annotate the corpus. Tutubalina et al. [6] finds that approximately 60% of mentions from each test set were also in the training set, suggesting an easy benchmark. Consequently, it proposes a third dataset named “*Custom CADEC*”, more popularized in [32], which is a fivefold cross-validation dataset (no development sets) avoiding redundancy between training and test sets. The mentions without an associated concept in the ontology as well as ambiguous mentions have also been removed.

The Corpus of Online Medical EnTities (COMETA): The COMETA corpus [40] consists of 20 K English biomedical mentions in layman’s language from an internet forum, and shares some similarities with all the CADEC datasets. A version of the whole SNOMED-CT is used as ontology, and almost half of the mentions are annotated by a concept from the “Clinical Finding”. There are two splits of this corpus: a “stratified split” in which, for each concept appearing in the test/dev sets, this concept appears at least once in the training set, and a “zero-hot split” in which there is no overlap between the concepts appearing in the test/dev sets and the train set.

##### Task 4 of adverse drug reaction extraction from drug labels (ADR) dataset from TAC 2017

The Text Analysis Conference^10^ (TAC) proposed a track to extract adverse reactions from drug labels [3]. Task 4 was an independent normalization task which uses the MedDRA ontology, which contains around 20,000 concepts. Originally, the task did not use accuracy measure, but nowadays, authors that evaluate their methods on this dataset use it preferably.

##### The chemical disease relation track of the BioCreative V challenge

This track was originally designed for joint entity recognition and normalization [41]. Two types of entities should be extracted and normalized: disease and chemical. The disease mentions are to be normalized with the MEDIC ontology and the chemical mentions by the non-hierarchical Comparative Toxicogenomics Database^11^ (CTD). Authors use now separated corpora, one for each entity type: the disease dataset (BC5CDR-D) and the chemical dataset (BC5CDR-C). The accuracy is also the preferred evaluation metric. Earlier, the BioCreative challenges have shared some others normalization datasets, such as the Gene Normalization datasets from BioCreative I [40], BioCreative II [42] and BioCreative III [43], but to our knowledge, they are more rarely used nowadays as gold standards.

##### The psychiatric treatment adverse reactions corpus (PsyTAR)

This annotated corpus [44] is also based on the AskaPatient forum and on annotations from the SNOMED-CT and the UMLS Thesaurus. It deals with effectiveness and adverse reactions of psychiatric medications. For evaluation purposes, Miftahutdinov and Tutubalina [32] proposed two datasets (each being a k-fold cross-validation dataset): a “*Random PsyTAR*” and a “*Custom PsyTAR*”, the latter avoiding the train/test redundancy.

##### Task 3 “normalization of adverse drug reaction mentions” dataset from SMM4H

The 2nd Social Media Mining for Health Applications^12^ (SMM4H) proposed an independent normalization task of adverse drug reactions in social media texts. The MedDRA ontology is used for annotations. All data sets are available.^13^

##### Task 1 dataset from the ShARe/CLEF eHealth evaluation lab 2013

The annotated mentions in this corpus are disorders. The original task is a joint entity recognition and normalization tasks [45]. A disorder is defined as any span of text which can be mapped to a concept in SNOMED-CT and which belongs to the Disorder Semantic Group from the UMLS. Although the clinical data in the corpus has been anonymized, the corpus is not open and requires some registration procedures to be manipulated.

##### The medical concept normalization (MCN) corpus

The fourth i2b2/VA shared task [46] proposed a corpus for entity recognition evaluation. Luo et al. [47] start from the full SNOMED CT and the RxNorm [48] ontologies to annotate the recognized mentions and build the Medical Concept Normalization (MCN) corpus. The mentions are disorders, as well as other medical problems, treatments and tests.

##### TwADR-L

This dataset^14^ [26] is composed of tweets that have been annotated by concepts from the SIDER 4 database of drug profiles.^15^ The used version of this database contains 2220 concepts. The corpus is separated into 10 folds for cross-validation.

#### Comparative evaluation

In specialized domains, there is no initiative to share entity normalization gold standards in a single place that would facilitate multiple evaluations and comparisons of proposed methods. Comparatively in the open domain, there are some interesting initiatives to compare entity disambiguation gold standards, such as the "*Disambiguate to Knowledge Base*" (D2KB) experiment from the GERBIL benchmarking system [33]. Rosales-Méndez et al. [49] suggests focusing on multilingual EL evaluation through a benchmarking set. van Erp et al. [50] shows some strengths and weaknesses of EL datasets, some are also interesting to EN (e.g. overlapping mentions between train and test sets). Sevgili et al. [51] compares some deep learning results of EL methods. One reason for this difference between specialized and open domains is that the access to clinical data (i.e. documents or knowledge bases) is often restricted [52].

Moreover, there are few detailed comparisons of features of normalization datasets in specialized domains. Corpus publications focus mainly on intrinsic information about the corpus used: the number of documents, sentences, words, mentions or used concepts in the full corpus. In particular, information on the separation into different folds, when this separation is done, is rarely provided. Most of the existing inter-analysis of datasets were done in publications which describe a new method evaluated on some datasets. Sometimes, the initial annotated corpus is not separated into different folds, and it is the authors of a method who publish a new dataset with specific sets for evaluation purpose. For instance, the “*Random CADEC*” dataset [26] is the result of the separation of the all-in-one CADEC corpus [39]. Note that during this process, some modifications can be made, such as simplifications (e.g. removing NIL or ambiguous mentions).

In [26], the average number of mentions that a concept annotates (considering only used concepts from the whole ontology) are presented for three datasets. Although not discussed, this information was possibly intended to give an idea of the difficulty of the benchmark: if low, the task will fall within more challenging few-shot or one-shot learning. In [53] or [11], in addition to the usual information but decoupled on the training set and the test set, the number of NIL mentions are also presented between the ShARe/CLEF, the NCBI-DC and the TAC-ADR datasets. For example, 32.7% of the test mentions in the ShARe/CLEF corpus are NIL mentions. A method that does not have a conceptless class in its prediction categories would therefore be at a disadvantage on this dataset. In [22], two distinct folds were created from a unique dataset where each one contains annotating concepts which are not in the other. One is chosen to train their method, the other for evaluation. This represents an analysis of the Zero-Shot Learning (ZSL) cases, that is, the mentions’ concept to predict have never been seen during training. In [54], the authors have clarified that some datasets do not use the whole ontology initially considered, but a subset built from the concepts contained in the training and test sets. They also present some ZSL indicators and the proportion of ambiguous mentions per dataset. The ZSL information is interesting because it gives an idea of the performance of a method on a real corpus. Indeed, in specialized domains, since the ontologies used for annotation contain a large number of concepts, it is futile to hope to have even one example for each concept in a gold standard. Tutubalina et al. [55] describes a cross-domain approach to extrinsically evaluate the ability to address zero-shot: by training a method on a dataset, then using it on another dataset and at inference time to estimate the nearest label for each mention.

To our knowledge, the first and unique work which directly concerns entity normalization comparative evaluation in specialized domains is [55]. The authors have analyzed five datasets (NCBI-DC, BC5CDR-D, BC5CDR-C, TAC-ADR and SMM4H) and have compared the NCBI-DC state-of-the-art method BioSyn [23] to a baseline based on BERT [56] on them. In particular, they observe in some datasets that a large number of mentions appear several times in the test set, and many are also seen at training time. This is also true for the Random CADEC dataset, and on refined test sets intended to reduce these biases. In fact, Tutubalina et al. [6] shows that the distribution of mentions through the folds have a significant impact on performances, leading to an average of 15% loss between initial and refined test sets.

### Contributions

- We propose indicators for characterizing EN datasets. We hypothesize that these indicators could help to categorize dataset for future work, regardless of language type or domain, but rather in terms of difficulties for current adaptable approaches.
- We propose a new mixed EN baseline method, which runs in a few minutes on a standard laptop (CPU). While not a strong baseline, it is a generic and easy-to-use one, whose performance well serves as an indicator of the difficulty of an EN task.
- We formalize an accuracy measure to evaluate EN methods, which takes into account the multi-normalization cases, which are often overlooked.
- We identify some potential biases of entity normalization evaluation: (1) Preprocessing may not be well described in the reference article of a method, while it may be domain-specific, and be responsible for the superior performance of a method. (2) Because all batches for some datasets are public, some evaluate on the subpart of the target ontology that contains only the concepts used in the test set. This can significantly (and arbitrarily) increases the performance of a method. (3) As soon as a method possibly predicts more than one concept per mention, the classical accuracy measure does not penalize wrong predictions. Moreover, in cases of multi-normalization, there may be several formulas or implementations, leading to different scores and thus to potential biased comparison.

## Methods

### Entity normalization methods

We report the leaderboards of the BB4 bacterial habitat task and the NCBI Disease Corpus in Tables 2 and 3 respectively. The main evaluation metric used for the BB4 task is a non-strict similarity score [19], but an accuracy metric is also provided by the task organizers through an evaluation web platform. For fair comparisons between datasets and methods, we only report here the results with accuracy as provided by BB4 task organizers and in [57] for the last published methods. This accuracy does not change the ranking of the first three methods compared to the similarity score, but has an impact on the next ones. To our knowledge, these two leaderboards represent the state-of-the-art in mid-2022.

**Table 2:** Accuracy of methods on the test set from the BB4 bacterial habitat dataset

Method	Accuracy
C-Norm [57]	60.4
HONOR [58]	53.1
CONTES [21]	50.0
PADIA BacReader [59]	48.8
BOUN-ISIK [60]	42.8
BLAIR GMU [61]	21.1
Baseline	22.4

The baseline is a DictB-SymR method used by task organizers that performs an exact match between lemmatized entity mentions and ontology concept labels

**Table 3:** Accuracy of methods on the NCBI-DC dataset

Method	Accuracy
BioSyn + (init. w/) SAPBERT [62]	92.5
[63]	92.1
[64]	91.7
Train:OD + Search:tuned [65]	91.1
BioSyn [23]	91.1
BCNH [66]	90.6
TripelNet [65]	89.9
Lightweight model [11]	89.5 ± 3.22
BERT-based ranking [53]	89.0 ± 3.32
TaggerOne [67] (from CNN-based ranking)	88.8 ± 3.32
NormCo [27]	87.8
BNE [68]	87.7
CNN-based ranking [69]	86.1 ± 3.63
Sieve-based [12]	84.7 ± 3.84
DNorm [70] (from CNN-based ranking)	82.2 ± 4.05

Confidence intervals (at a confidence level of 0.05) come from [11]

To support our purposes, we have chosen the state-of-the-art method on each corpus at the time we started our experiments in 2020, that is C-Norm [57] for BB4 and BioSyn [23] for NCBI-DC. To our knowledge, no previous publication has shown evaluation of C-Norm on the NCBI Disease Corpus, or of BioSyn on the BB4 dataset. BioSyn and C-Norm have the advantage of being at least publicly shared, but it is not yet a widespread effort in the entity normalization community. Our experience shows that the published results are mainly reproducible. Nevertheless, we have not conducted additional experiments to study reproducibility issues (e.g. non-significant scores). We also created a baseline that basically combines the four strategies (i.e. DictB, DistR, SymR and ML), each in their naive version.

#### BioSyn

BioSyn [23] was the state-of-the-art method on the NCBI Disease Corpus dataset at the time we started our experiments in 2020. It’s a ML-based method using TF-IDF bag-of-word representations [71] (i.e. DictB approach) as well as embeddings (i.e. DistR approach) of mentions computed by BioBERT [72]. The method uses both representations to predict top-k candidates by weighting the influence of one or the other in a final score, that is, by weighting the influence of morphological or distributional information. During training, BioBERT embeddings are fine-tuned to modify this score. Since concepts regularly have multiple labels, training is done by seeking to maximize the probabilities of predicting all the correct concept labels in the top-k candidates for each mention, not only the top-1 as most methods do. During the training, the system learns to rank k candidates containing also some negative examples with high probability of prediction. The authors claim that this kind of integration of negative sampling allows to improve the BioBERT embeddings for the task.

Even if not described in detail in [23], the method includes some preprocessing: lowercasing and punctuation removal of mentions and labels, an acronyms resolution with Ab3P,^16^ some rules to resolve composite mentions and a list of corrections for bad spelling of some words.

We used the official available code^17^ for preprocessing and the core method.

#### C-Norm

C-Norm [57] is the current state-of-the-art method on the BB4 dataset. It’s a DistR-ML which uses a weak supervision strategy (i.e. use of all label-concept couples from the ontology as positive examples) and integrates some ontological information. The method does not represent each concept through their labels, but by a unique semantic encoding vector which is mainly based on the subsumption information available in ontologies. The method learns a projection from an embedding space to an ontological space defined by the semantic vectors of concepts. The objective function tries to globally maximize the cosine similarities between projected vectors of mentions and their concept vector(s).

C-Norm uses as input Word2Vec embeddings which should be fine-tuned in a specific way, including preprocessing (lowercasing, lemmatization and stopword removal) and a training corpus selection.

We have encountered difficulties in running the method on corpora with larger ontologies and/or datasets. We have optimized the code so that it can more easily run on a Google Colab CPU server^18^ (limited to 12Go RAM). One of the hyperparameters of the method is the number of convolutional filters. The authors state that it should be set to the number of concepts in the used ontology. On the NCBI Disease Corpus, we fixed that number to 3000 as on the BB4 dataset, rather than 9664 as it should be (i.e. there are 9664 concepts in the MEDIC ontology). Finally, we don’t exactly use the scripts pointed by the authors, but instead reproduce the preprocessing steps. Indeed, these preprocessing should be performed through the corpus processing engine AlvisNLP/ML,^19^ which made it difficult to use on our server. Despite our modifications, we found back similar results on the BB4 corpus to those published.

#### Baseline

As we mentioned, baseline methods are often absent from normalization studies. D’Souza and Ng [12] describes the first sieve approach for entity normalization. This approach is based on several methods which are sorted by expected precision, and inversely, by expected recall. The most precise method is used first on a dataset, and the mentions that do not have predictions are then passed to the second most precise method, and so on. The first sieve method was based on DictB-SymR methods, but Ferré et al. [58] proposed a 2-sieves method where the second sub-method is based on DistR-ML and trained on the whole training set.

Inspired by this approach, we construct a simple baseline based on two sub-methods: a first DictB-SymR method, and a second DistR-ML one. This combined approach aims to leverage the strengths of each type of classical approach, and, rather than being a strong baseline, it is expected to be robust to domain or task changes. The DictB-SymR method starts by adding mentions of a training dataset as labels of the associated concept, as it is also done by BioSyn. Unlike BioSyn, if several mentions with the same surface form are annotated by different concepts, the mention is added as a label only for the concept that was the most frequent. Then for prediction, the method lowercases and stems all mentions and all labels and seeks for exact match. In a very simplified way, by adding training examples, this method is also ML-based. The DistR-ML method uses standard biomedical embeddings,^20^ and is trained to project all mention embeddings (i.e. the vector averaging the embeddings of the tokens from the mention) near the label embeddings of their concept. The learned projection can then be used on prediction, where the predicted concept is the one which has a label embedding nearest to the projected mention embedding. Other representation models have been proposed that we could have used, such as BERT [56], specialized models such as SapBERT [62] for the biomedical domain, and TF-IDF representations [73]. This is left as future work. We stress that our baseline has the advantage of being low on RAM consumption and runs in a couple of minutes on a standard laptop, and is not specific to a particular domain.

### Dataset description

For this work, we particularly focus on two datasets, which have in common to be frequently used to evaluate methods: the BB4 and the NCBI-DC datasets. Both are publicly available and use a relatively small and hierarchically organized ontology. For BB4, the bacterial habitat hierarchy of the OntoBiotope ontology owns 3172 concepts. For the NCBI-DC, the MEDIC ontology owns 9664 concepts. Due to the hierarchical nature of these ontologies, there are no NIL mentions (i.e. each mention of interest can be at least normalized by the root concept).

The full BB4 dataset has three entity types (habitat, phenotype and microorganism), but as observed in [57]: “*the Phenotype part of the dataset is much smaller than the Habitat part and thus it is harder to observe clear tendencies on this dataset*”. Moreover, the microorganism part is not really challenging regarding terminological variations, and the ontology used for this part is relatively large. Thus, as others do, we only use the habitat part of the dataset. Nevertheless, for some experiments, we use the full OntoBiotope ontology, which also owns a phenotype hierarchy with 429 concepts.

The NCBI-DC has a particularity: the presence of unresolved composite mentions (e.g. the composite mention “*breast or ovarian cancer*” is given as it is, rather than resolved in two mentions: “*ovarian cancer*” and a discontinuous mention “*breast cancer*”). The objective of the task is then to normalize these mentions by all the concepts that should normalize each of their component entities. We choose to consider these mentions as any other case of multi-normalization.

For comparison purposes, we analyzed some indicators on the “*Custom CADEC*” dataset. This dataset is different from NCBI-DC and BB4: it is separated into five-folds for cross-validation, their annotating ontologies are relatively big and it has been already reworked from its previous version "*random CADEC*" to correct some observed biases. Moreover, the corpus is publicly shared, but not the two ontologies. The Clinical Finding hierarchy of the SNOMED-CT owns 99,814 concepts, and it is unclear what is the part of the non-hierarchical AMT terminology which has been used (the trade and medicinal product types mainly used are 7188).

### Our proposal for a quality measure

As discussed, there is no standard evaluation system for the NCBI Disease Corpus. Authors typically resort to their own scripts for computing accuracy, without describing it upon mentioning “we used accuracy”. Thus, the evaluation of multi-normalization cases is then not explicit. For instance with BioSyn, the personal accuracy evaluates multi-normalization cases, but differently depending on whether they are composite mentions (all concepts are needed to validate correct prediction) or multi-labeled mentions (just one of the correct concepts is needed to validate correct prediction).

To our knowledge, no formalization of an accuracy measure taking into account the multi-normalization cases has yet been proposed (although we believe that such a measure may have already been implicitly used in some tasks). The measure is detailed in Eq. 3:

3 $$Acc = \frac{1}{N} \cdot \mathop \sum \limits_{i = 1}^{N} \frac{{\mathop \sum \nolimits_{j = 1}^{{p_{i} }} strict\left( {m_{i} , c_{i}^{j} } \right)}}{{n_{i} }},$$

where $strict\left( {m,c} \right) = 1$ if c is a correct concept of m, 0 otherwise

Equation 3: Equation of accuracy generalized to multi-normalization cases. Here $p_{i}$ is the number of predicted distinct concepts $c_{i}^{j}$ for the mention $m_{i}$, and $n_{i}$ is the correct number of distinct concepts to find.

As the NCBI-DC dataset has the specificity of containing some unprocessed composite mentions, which are evaluated as in Eq. 3, in addition to the cases of multi-labeled mentions, the BioSyn method proposes a slightly different evaluation script: a method only needs to predict one of the correct concepts to get the full point for a multi-labeled mention. This seems to us as relatively mild compared to Eq. 3. As a contribution, we therefore propose a generalized accuracy in Eq. 4. In particular, the denominator does not involve only the number of correct concepts to predict as in Eq. 3, but also the number of predicted concepts. Our measure introduces indeed a penalty (i.e. here with a choice among others of a max operator) in case a method would predict more concepts than expected. Indeed, if a method predicts all the concepts of the ontology for each mention, and that it only counts to predict the correct concepts, it would get a perfect score. We think that the reason for this unaddressed problem in the classic accuracy comes from the fact that the majority of existing methods propose a single prediction per mention.

4 $$Acc = \frac{1}{N} \cdot \mathop \sum \limits_{i = 1}^{N} \frac{{\mathop \sum \nolimits_{j = 1}^{{p_{i} }} strict\left( {m_{i} , c_{i}^{j} } \right)}}{{max\left( {n_{i} ,p_{i} } \right)}},$$

where $strict\left( {m,c} \right) = 1$ if c is a correct concept of m, 0 otherwise.

Equation 4: Our general equation of accuracy. Here $p_{i}$ is the number of predicted distinct concepts $c_{i}^{j}$ for the mention $m_{i}$, and $n_{i}$ is the correct number of distinct concepts to find.

We also use this accuracy function on the BB4 corpus, which seems to give the same results as the one used on the BB4 online evaluation platform.^21^ Note that if a method predicts a single concept per mention in a multi-normalization dataset, then Eq. 4 reduces to Eq. 3.

### Indicators for comparison

To be capable of comparing datasets, we choose some general indicators, including some which have never been used in our context. We categorize these indicators as “*intra-folds*” and “*inter-folds*”.

#### Intra-folds indicators

As in [26], we compute the average number of mentions that a concept annotates (considering only used concepts from the whole ontology). As in [53] or [11], we analyze the number of NIL mentions, and we choose to use the percentage of NIL mentions to enable comparisons. Finally, as in [54], we compute the percentage of ambiguous mentions, that is, the percentage of mentions with the same surface forms that are annotated by different concepts. We also show the percentage of multi-normalization cases, that is, the mentions which should be normalized by two or more distinct concepts (including composite mentions in NCBI Disease Corpus).

#### Inter-folds indicators

As observed in [6] and in [55], some datasets have a high overlap of mentions between training and test sets. This could lead to relatively optimistic results. Thus, we compute a redundancy indicator, which is the percentage of examples (i.e. not the mentions, as in other studies) seen in the training set and seen again in the test set. Conversely, we compute a ZSL indicator (i.e. for Zero-Shot-Learning) which is the percentage of concepts met in the test set but never in the training set. For instance, the “zero-shot split” of the COMETA corpus has a ZSL of 100%: no concept of the test set is seen in the training material. These ZSL cases are considered challenging, and we think that the proportion of these in a dataset can give an estimation of the difficulty of a task.

## Results

### Intrinsic analysis of corpora

Our first aim is to illustrate the difference between the NCBI Disease Corpus and the BB4 datasets. If they are too similar, it should be difficult to estimate the robustness of a method by only analyzing their results on these two datasets. To measure this difference, we use our intra and inter-folds indicators previously introduced.

#### Intra-folds

We report in Table 4 that the BB4 and NCBI-DC corpora are both realistic representatives of a Few-Shot Learning (FSL) corpus, with half of the concepts in the training set annotating only one or two distinct mentions (FSL_median_ = 2). Compared to them, the Custom CADEC corpus seems to offer many more distinct examples of mentions per concept, with half of the concepts annotating at least 6 distinct mentions. Note that there is an important imbalance in the number of examples per concept, whatever the corpus: there are some concepts with dozens of distinct mentions and many with only a few.

**Table 4:** Few-shot learning (FSL) indicators, that is, the average (or median) of mentions annotated by concepts in the training set

	BB4 (train)	NCBI-DC (train + dev)	Custom CADEC (average on all train folds)
FSL_average_	5.1	8.8	9.5
FSL_median_	2	2	6.4
FSL_max_	133	235	59
FSL_average_ (distinct)	3.1	2.9	9.5
FSL_median_ (distinct)	2	1	6.4
FSL_max_ (distinct)	34	61	59

Only the concepts that annotate at least one mention are considered. The “distinct” indicators mean that mentions with the same surface form contribute only for one example

We show in Table 5 that the BB4, NCBI-DC and Custom CADEC corpora can be differentiated by the percentage of multi-normalization cases and the percentage of ambiguous mentions. Notably, BB4 has 8.1% of multi-normalization cases in its training set. If the test set has a similar ratio, this would result in at least 4 points less in accuracy for methods which do not address this problem. In contrast, as indicated in [32], all multi-normalization cases (called “*ambiguous mentions*” in the study) have been removed from the Random CADEC corpus to build the Custom CADEC corpus.

**Table 5:** Multi-norm cases are the mentions which should be normalized by two or more distinct concepts (including composite mentions in NCBI Disease Corpus)

	BB4 (train + dev) (%)	NCBI-DC (train + dev + test) (%)	CADEC custom (all folds) (%)
Multi-norm	8.1	2.3	0.0
NIL	0.0	0.0	0.0
Ambiguity	19.3	4.6	1.2

Ambiguity is the percentage of mentions with same surface forms that are annotated by different concepts through the considered corpus. NIL (not in lexicon) is the percentage of mentions which should not be normalized by any concept in the reference (often normalized by a “CONCEPT_LESS” label)

The most notable difference between the three datasets is the rate of ambiguous mentions. Indeed, while NCBI-DC and Custom CADEC contain a low ambiguity rate (less than 5%), almost one in five BB4 mentions is ambiguous. To resolve those ambiguities, it would be necessary to use the context of the mentions, but to our knowledge, this information is never used by existing methods in entity normalization (at the difference of entity linking methods).

It is noteworthy that in the three datasets, there are no NIL mentions. The hierarchy of the OntoBiotope and MEDIC ontologies means that all mentions can at least be normalized by their root. Nevertheless, in practice, there are no mentions normalized by the root in these datasets: a NIL mention could be resolved by completing the ontology with a more specific concept during the corpus building (e.g. it was the case for the BB4 dataset). Moreover, a direct mention in text of a root concept (e.g. “*bacterial habitat*”) can usually be considered as too uninformative to be extracted. Finally, the NIL mentions can simply be deleted from the corpus. For instance, because of the non-hierarchical structure of the AMT part of the Custom CADEC dataset, there are NIL mentions in the initial CADEC corpus. But these ones have been deleted when building the Custom CADEC dataset.

#### Inter-folds

As shown in Table 6, redundancy and zero-shot-learning indicators can also clearly differentiate the three corpora. As redundancy was a major bias in the Random CADEC corpus, we observe indeed that there is nothing left in the Custom CADEC. The authors were able to obtain 5 folds (rather than 10 initially) by redistributing examples (seeming to duplicate examples from the initial corpus between batches) and obtain only 181 used concepts (rather than 1036 initially). This modification could also explain the higher number of distinct examples by concepts observed in Table 4. Compared to this, the NCBI Disease Corpus displays a high rate of redundancy (63.3%), close to the initial rate of the Random CADEC dataset. Because of their relatively low rate of ambiguities (see Table 5), redundancy appears also as a major biais in the NCBI Disease Corpus. The redundancy is lower in the BB4 dataset (24.6%).

**Table 6:** Redundancy is the percentage of examples met in the training set and met again in the test set

	BB4 (train on dev) (%)	NCBI-DC (train + dev on test) (%)	Custom CADEC (average on all train on test folds) (%)
Redundancy	24.6	63.3	0.0
ZSL	48.2	27.3	0.0

ZSL (zero-shot learning) is the percentage of concepts met in the test set and never met in the training set

ZSL is also an interesting indicator to differentiate the datasets. Notably, although Custom CADEC addressed the redundancy problem, it keeps a distribution of examples between sets, which results in a perfect overlap between concepts used in the training sets and their associated test sets. Compared to this one, around a quarter of concepts to predict in the test set have never been used in the training set of the NCBI Disease Corpus, and around a half for the BB4 dataset. Without judging the relevance of having a strong ZSL value in an evaluation corpus, a null value does not allow to evaluate the capacity of a system to predict a concept it has never seen.

### Significativity/variability of methods

A screening of all methods’ parameters has a significant cost in time. This time needs to be multiplied to estimate the significativity and the variability (due to stochastic parts in numerous methods) of the results. These efforts are particularly important when the aim is to compare different methods that obtain very similar scores on the same dataset. This may be the reason why some authors do not test for significance and present only one result in their publication (e.g. BioSyn). This represents a bias when we try to unknowingly compare a method that would give a significant score with a method that would give its best score, enough to change a leaderboard. Nevertheless, in this study, we seek to observe important differences. Based on the articles of the BioSyn and the C-Norm methods, and on our tests, we assumed in our experiments that a difference of 3 points is significant enough to make observations on single runs, regardless of the modifications made.

### Influence of preprocessing and used resources

We evaluate the impact of preprocessing on the performance of the methods. If a preprocessing gives a positive impact on a dataset, not on another one, then it reveals that these preprocessing are somehow domain-specific, thus lacking robustness. We think that a realistic and non-domain-specific baseline method can also help to characterize a corpus. This baseline serves as a control through different datasets. Rather than only measuring the score of a method alone on a dataset, observing the difference between this score and that of the baseline should give a better appreciation of the quality of the results.

#### BioSyn

For BioSyn, it should be noted that score variation doesn’t come from training (with same BioBERT version at least), but only from preprocessing. As shown in Table 7, the whole preprocessing seems to have a marginal influence on the accuracy of the method on the BB4 dataset (+ 2.5 points), and has even a slightly negative influence on our baseline method (− 1.3 points). On the other hand, preprocessing has a global positive influence on the accuracy on the NCBI-DC dataset (+ 8.3 points). It seems to indicate that preprocessing is globally over-specialized to the NCBI-DC dataset. However, on a single run, it is difficult to estimate the real individual contribution of each preprocessing option (maximum observed contribution of 2.8 points). Surprisingly, it can be noted that removing the typo resolution, though specialized to the typos contained in the NCBI-DC corpus, gave a slightly higher score (+ 0.6 points) for the method with all the preprocessing. Thus, it is not a significant observation, but it is enough to question the relevance of this preprocessing which would require important manual efforts while being strongly domain-specific.

**Table 7:** Ablation study of BioSyn preprocessing on the two datasets

	BB4 train/dev	NCBI-DC train + dev/test
Baseline	55.4	78.7
Baseline + BioSyn preprocessing	54.1	82.9
BioSyn	59.1	89.6
BioSyn (without any preprocessing)	56.6	81.3
BioSyn—lowercasing and punctuation removal	57.9	88.4
BioSyn—acronym resolution	57.0	86.8
BioSyn—composite mention resolution	57.3	88.4
BioSyn—typo resolution	57.8	90.2

We use our accuracy metric (see Eq. 4) rather than the one used by the BioSyn method. The “X/Y” means that training was performed on X and evaluation on Y

We also observe a huge gap between the results of BioSyn on the two datasets: + 30.5 points on the NCBI-DC dataset compared to the BB4. We have a similar observation with our baseline method (+ 23.3 points) or with our baseline with BioSyn preprocessing (+ 28.8 points). The difference of measures presented earlier between the two corpora may explain this difference. Moreover, from our observations, BB4 ambiguous cases in particular lead BioSyn to predict too many concepts. And finally, we show that BioSyn outperforms our baseline, with or without BioSyn preprocessing (+ 3.7 compared to the baseline on BB4, and + 6.7 to the baseline with BioSyn preprocessing on the NCBI-DC).

#### C-Norm

In Table 8, we compare the impact of BioSyn preprocessing on the results of C-Norm. Surprisingly, C-Norm does not seem to gain any particular benefit from the NCBI-DC over-specialized BioSyn preprocessing. Thus, the method seems relatively robust and can mitigate the need of these kinds of preprocessing.

**Table 8:** Accuracy of C-Norm (with 3000 filters) on the both datasets, with and without using the preprocessing of BioSyn on the data

	BB4 (train on dev)	NCBI-DC (train + dev on test)
C-Norm	64.8	73.8
C-Norm + BioSyn preprocessing	65.0	74.3

BioSyn preprocessing are lowercasing, punctuation removal, acronym resolution with Ab3P, and handmade typo resolution (for NCBI-DC dataset). Composite mention resolution was not applied on the BB4 dataset because the composite mentions were already resolved

Despite the suboptimal use of only 3000 convolutional filters on the NCBI-DC dataset, we can still observe a gap between the results of C-Norm on the two datasets: + 9.0 points on the NCBI-DC dataset compared to the BB4.

C-Norm applies as preprocessing only lowercasing, lemmatization and stopword removal and no strongly domain-specific preprocessing is used. But on the contrary, C-Norm is based on a specialized Word2Vec embedding set, optimized directly on the BB4 development set with another normalization method [17]. As shown in Table 9, the use of a standard embedding set (even specialized for the biomedical domain and trained on the same type of literature) has a negative impact on the C-Norm results: − 5.9 points on the BB4 and − 6.8 points on the NCBI-DC. A similar observation can be done with our baseline method on the BB4 (− 3.4 points) but not on the NCBI-DC (+ 6.4 points). For C-Norm, which is strongly based on word embeddings, the main explanation could be the number of out-of-vocabulary tokens. Indeed, 31.7% of tokens from NCBI-DC dataset and MEDIC terminology are out-of-vocabulary in the BB4 embedding set. The DictB-SymR submethod of our baseline, which is not based on embeddings, could explain why this one performs better.

**Table 9:** Accuracy of C-Norm (with 3000 filters) and our baseline on the both datasets, with custom embeddings and more standard embeddings

	BB4 (train on dev)	NCBI (train + dev on test)
Baseline (with standard biomedical embeddings)	55.4	78.7
C-Norm (with standard biomedical embeddings)	58.9	67.0
Baseline + C-Norm embeddings	58.8	72.3
C-Norm	64.8	73.8

The “X on Y” means that training was performed on X and evaluation on Y

As noted, a minimized value of the number of filters seems to have a real negative impact on C-Norm scores on NCBI-DC. But from a reproducibility point of view, our use of both methods gives accuracy scores close to the ones published:

- For BioSyn on the NCBI-DC: 89.6 with our run on the test set, and 91.1 for the run in the publication.
- For C-Norm on the BB4: 64.8 with our run on the development set (with other sentence/word segmenters), and 63.3 for the average run in the publication (standard deviation of 0.9).

### Influence of reference size

As noticed in [54], some datasets use a subpart of the initial reference ontology. We analyze the impact of choosing different parts of the ontology. Mainly, we built smaller ontologies in keeping only the concepts used in train and/or test sets. For OntoBiotope, we also use the whole ontology with the phenotype part.

As shown in Table 10, the BB4 dataset used in our experiment contains 3172 concepts of bacterial habitats (88%) out of the 3602 of the whole OntoBiotope ontology. The habitat part is the main part of OntoBiope. Compared to that, the train + dev dataset represents only 294 concepts (8% of the whole, 9% of the habitat part).

**Table 10:** Number of concepts considered in some subparts of the OntoBiotope ontology for the BB4 task

	Full OBT	Habitat OBT	Train + dev habitat OBT	Train habitat OBT
Number of concepts	3602	3172	294	232

As shown in Table 11, these differences of the possible target concepts have a strong influence on the results on the dev set for C-Norm (+ 9.2 points). The gain is less visible for BioSyn (+ 2.7 points) and our baseline (+ 3.8 points). Certainly because of the relatively low number of overlaps between used concepts in the train and the dev (51.8%, see ZSL indicator in Table 6), all the methods strongly decrease their results when using only the concepts from the train (− 14.1 points for C-Norm, − 16.5 for BioSyn and − 12.6 for our baseline). We also observe that C-Norm has a slight loss when using the whole ontology (− 1 point), notably compared to BioSyn (− 5.6 points). Thus, C-Norm seems more robust to concepts addition than BioSyn on the BB4 dataset.

**Table 11:** All methods are trained on training dataset and evaluated on dev dataset

	Full OBT	Habitat OBT	Train + dev habitat OBT	Train habitat OBT
C-Norm	63.8	64.8	74.0	50.7
BioSyn	53.5	59.1	61.8	42.6
Baseline	53.9	55.4	59.2	42.8

For train + dev, train and dev versions of OntoBiotope, we do not reorganize the broken subsumption hierarchy

With the availability of the annotated test set of the NCBI-DC dataset, the methods can use more different subsampled ontologies. We do not use the MEDIC ontology with only the concepts used in the test set, which would be borderline, as the dev Habitat Ontobiotope for BB4. As shown in Table 12, the NCBI-DC dataset used in our experiment contains 9664 concepts, with the train + dev + test dataset representing only 751 concepts (8%) and the train + dev dataset only 695 (7%). There is thus a certain similarity of distribution between the concepts used in the annotated data and all the predictable concepts.

**Table 12:** Number of concepts considered in some subparts from MEDIC for the NCBI Disease Corpus

	Full MEDIC	Train + dev + test MEDIC	Train + dev MEDIC
Number of concepts	9664	751	695

As shown in Table 13, the use of the train + dev + test MEDIC has no visible influence on the results of BioSyn (+ 1.2 points), and a weak gain for our baseline (+ 3.2 points). In contrast, it has a strong influence on C-Norm (+ 12.5 points). Thus, we can observe here a similar behavior of the three methods whether on the BB4 or on the NCBI-DC datasets. Because of a higher number of overlaps between used concepts in the train + dev and the test (72.7%, see ZSL indicator in Table 6), the effect of using only the train + dev concepts seems to be less drastic (− 10.4 points for BioSyn and − 5.6 points for our baseline), with C-Norm even obtaining a little gain (+ 2.4 points). Therefore, C-Norm seems to better benefit from concepts filtering which are not in the set of possible predictions.

**Table 13:** All methods are trained on train + dev dataset and evaluated on test dataset

	Full MEDIC	Train + dev + test MEDIC	Train + dev MEDIC
C-Norm	73.8	86.3	76.2
BioSyn	89.6	90.8	79.2
Baseline	78.7	81.9	73.1

For train + dev + test and train + dev, we do not reorganize the broken subsumption hierarchy

### Influence of scoring metric

The BioSyn script proposes a scoring evaluation with different features than ours (see Eq. 3). Notably, it evaluates multi-labeled mentions differently: if the predicted concept is in the list of correct concepts, it is considered as a good prediction (i.e. maximum points for the current mention). With our metric, a good prediction means all expected concepts are predicted. From Table 14, we see that this difference has not a significant impact on the NCBI-DC dataset (0.1 point variation for our baseline, 0.4 for C-Norm and 0.6 point for BioSyn). The reason is that there are only 2.3% of multi-normalization cases in the NCBI-DC (see Table 5), and therefore even fewer multi-labeled mentions. On the contrary, for the BB4 dataset, there are exactly 8.1% multi-labeled mentions in the train + dev dataset (see Table 5). And we can indeed observe that with the BioSyn accuracy, there are significantly better scores for C-Norm (+ 3.9 points) and BioSyn (+ 6.5 points). It is less visible for our baseline (+ 1.9 points).

**Table 14:** Comparison of score calculated with our proposed accuracy or with the proposed BioSyn accuracy

	BB4 with our accuracy	BB4 with BioSyn accuracy	NCBI-DC with our accuracy	NCBI-DC with BioSyn accuracy
C-Norm	64.8	68.7	73.8	74.2
BioSyn	59.1	65.6	89.6	89.7
Baseline	55.3	57.2	78.7	79.3

Therefore, this experiment shows that it is possible to have a biased scoring metric. Without clarification and caution on the scoring measure used, a reported evaluation could in fact be misleading. In our experiment on BB4, we show that BioSyn could have been used to present a higher accuracy than C-Norm (65.6 points vs. 64.8 points).

## Discussion

To better evaluate the robustness and the adaptability of methods, evaluation on distinct datasets are needed. Nevertheless, on the existing datasets, some have no publicly available resources (e.g. the specific version of the SNOMED CT-AU annotating part of the CADEC datasets is not open source, and not free for everyone). This is an obstacle for the reproducibility of the work. In this way, the work described in this article could be extended to more datasets and more methods. The community should also make efforts to produce datasets from outside the biomedical (or biological) domain. An open source warehouse with many datasets could support this purpose.

A personal evaluation system seems to us to be a significant source of possible errors. Notably, we showed that it can induce a bias if there are differences in the scoring measure used. The full access to the annotated test set can also induce a bias by enabling some concepts filtering in the ontological reference making the task easier and/or less real world oriented. For these reasons, an independent online evaluation platform, similar to the one used by the Bacteria Biotope tasks, seems to be essential. Nevertheless, the unavailability of the annotated test set limits the possibility of discussions (e.g. on possible errors of annotation, on comparison of mentions/concepts distribution between train/dev dataset and test set or on designing k-fold cross-validation). By the way, for significativity purposes, k-fold cross-validation should become a standard, as well as a ZSL evaluation, notably to better apprehend the capacity of a method to address real world tasks.

We were surprised by the number of recently published methods that do not even share their code. But without taking into account this bad practice, there can also be a usability problem with the methods sharing their code. For instance, C-Norm needs a lot of computing resources, which means that even if it can be easily installed, it is not very usable without having access to a suitable computer. Compared to C-Norm, BioSyn seems much more usable (as we could run it on a free Google Colab GPU server).

A standardization of the normalization task could be a step forward for reproducible work and fairer comparison. For instance, some datasets already resolve the composite mentions in the provided dataset (e.g. BB4) while some others do not (e.g. NCBI-DC). Some datasets use a conceptless concept (e.g. CADEC), some others remove them (e.g. Custom CADEC) and some others give a hierarchical ontology which means that any mention can at least be normalized by the root concept (e.g. BB4). Some datasets are designed with some simplifications (e.g. ambiguous mentions have been removed for Custom CADEC). Some datasets had an intervention on the distribution of the examples (e.g. removing duplicate mentions in Custom CADEC). And as shown previously, there are some distinct accuracy functions. On this subject, our recommendations are:

- Separate into two tasks the resolution of composite mentions and the entity normalization.
- Promote hierarchical ontology rather NIL concept. Minimally, a list of concepts can be attached to a root that represents the entity type (i.e. the type of entity targeted by the prior recognition).
- Avoid simplifying datasets, and instead intervene to make them more realistic. In particular, redundancy should be minimized and ZSL cases should be amplified, because of the performance biases. Separate evaluations (e.g. cases of multinorm, redundancy, ZSL and ambiguity) could also help to estimate the strengths and weaknesses of methods.

What is often missing is a comparison with a good baseline that is relatively robust. This would allow to estimate the difficulty of a dataset, and also to estimate a bit better the real performance of a method by observing the difference from it. Ideally, such a baseline should be openly shared and easily manipulable. The baseline we have proposed here, if efforts to make it more easily usable were produced (e.g. by integrating it into a standard Python library), seems to us to meet this need. It also has the advantage of being able to perform a training phase and a prediction phase in only a few minutes.

We expect to improve our analysis by expanding our experiment to some other entity normalization datasets and methods in future work, and to study in more depth the performance of the methods according to the different cases mentioned.

## Conclusions

Even if some methods reach scores higher than 90 points of accuracy on some entity normalization datasets, we show that there exists some evaluation bias that renders comparisons between methods hazardous, and tends to distort the real performance of the state of the art methods. We hope that these biases will be taken into consideration by the entity normalization community, notably by the dataset/method providers. We were able to make some suggestions that could avoid these biases and therefore considerably support a methodological research on entity normalization.

## Data Availability

We conduct our experiments on free Google Colab servers (CPU or GPU), limited to 12 Gb of memory. All the code used for this work are available at: https://github.com/ArnaudFerre/data-norm. The code of the BioSyn method is available at: https://github.com/dmis-lab/BioSyn. Our modified code of the C-Norm method is available at: https://github.com/ArnaudFerre/C-Norm_PostLab. The NCBI Disease Corpus is available at: https://www.ncbi.nlm.nih.gov/CBBresearch/Dogan/DISEASE/. The Bacteria Biotope 4 dataset is available at: https://2019.bionlp-ost.org/. The biomedical embeddings file “PubMed-w2v.bin” is available at: http://bio.nlplab.org/.

## Abbreviations

EN: Entity normalization
NIL: Not-in-lexicon (or not-in-list)
DictB: Dictionary-based
DistR: Distributional representations
SymR: Symbolic rules
ML: Machine learning
BB4: (Entity normalization dataset from) Bacteria biotope 4
NCBI-DC: NCBI Disease Corpus
ZSL: Zero-shot-learning
FSL: Few-shot learning

## Footnotes

1 https://2019.bionlp-ost.org/.

2 https://tinyurl.com/OntoBiotope2019.

3 http://bibliome.jouy.inra.fr/demo/BioNLP-OST-2019-Evaluation/index.html.

4 https://www.nlm.nih.gov/mesh/meshhome.html.

5 https://www.ncbi.nlm.nih.gov/omim.

6 https://www.askapatient.com/.

7 A NCTS license is needed to obtain the ontologies. Contact the support team to obtain the former versions used to annotate the corpus (australianhelp@digitalhealth.gov.au).

8 https://www.meddra.org/.

9 https://zenodo.org/record/55013.

10 https://tac.nist.gov/.

11 http://ctdbase.org/.

12 https://healthlanguageprocessing.org/sharedtask2/.

13 https://data.mendeley.com/datasets/rxwfb3tysd/1.

14 https://zenodo.org/record/55013.

15 http://sideeffects.embl.de/.

16 https://github.com/ncbi-nlp/Ab3P.

17 https://github.com/dmis-lab/BioSyn.

18 https://colab.research.google.com/.

19 https://bibliome.github.io/alvisnlp/.

20 the “*PubMed-w2v.bin*” file from http://bio.nlplab.org/.

21 http://bibliome.jouy.inra.fr/demo/BioNLP-OST-2019-Evaluation/index.html.
